# Endogenous Expression of the Human CD83 Attenuates EAE Symptoms in Humanized Transgenic Mice and Increases the Activity of Regulatory T Cells

**DOI:** 10.3389/fimmu.2019.01442

**Published:** 2019-06-25

**Authors:** Elisabeth Zinser, Ronald Naumann, Andreas B. Wild, Julia Michalski, Andrea Deinzer, Lena Stich, Christine Kuhnt, Alexander Steinkasserer, Ilka Knippertz

**Affiliations:** ^1^Department of Immune Modulation, Universitätsklinikum Erlangen, Erlangen, Germany; ^2^Transgenic Core Facility, Max Planck Institute of Molecular Cell Biology and Genetics, Dresden, Germany

**Keywords:** CD83, transgenic mice, EAE, dendritic cells, B cells, T cells, regulatory T cells

## Abstract

The CD83 is a type I membrane protein and part of the immunoglobulin superfamily of receptors. CD83 is involved in the regulation of antigen presentation and dendritic cell dependent allogeneic T cell proliferation. A soluble form of CD83 inhibits dendritic cell maturation and function. Furthermore, CD83 is expressed on activated B cells, T cells, and in particular on regulatory T cells. Previous studies on murine CD83 demonstrated this molecule to be involved in several immune-regulatory processes, comprising that CD83 plays a key role in the development und function of different immune cells. In order to get further insights into the function of the human CD83 and to provide preclinical tools to guide the function of CD83/sCD83 for therapeutic purposes we generated Bacterial Artificial Chromosomes (BAC) transgenic mice. BACs are excellent tools for manipulating large DNA fragments and are utilized to engineer transgenic mice by pronuclear injection. Two different founders of BAC transgenic mice expressing human CD83 (BAC-hCD83^tg^ mice) were generated and were examined for the hCD83 expression on different immune cells as well as both the *in vitro* and *in vivo* role of human CD83 (hCD83) in health and disease. Here, we found the hCD83 molecule to be present on activated DCs, B cells and subtypes of CD4^+^ T cells. CD8^+^ T cells, on the other hand, showed almost no hCD83 expression. To address the function of hCD83, we performed *in vitro* mixed lymphocyte reactions (MLR) as well as suppression assays and we used the *in vivo* model of experimental autoimmune encephalomyelitis (EAE) comparing wild-type and hCD83-BAC mice. Results herein showed a clearly diminished capacity of hCD83-BAC-derived T cells to proliferate accompanied by an enhanced activation and suppressive activity of hCD83-BAC-derived Tregs. Furthermore, hCD83-BAC mice were found to recover faster from EAE-associated symptoms than wild-type mice, encouraging the relevance also of the hCD83 as a key molecule for the regulatory phenotype of Tregs *in vitro* and *in vivo*.

## Introduction

In recent years bacterial artificial chromosome (BAC) technology has offered new possibilities for the development of transgenic model systems in basic immunology to study complex immunological processes of murine and human genes in a more physiological context. BACs, on average 100–300 kb large in size, encode usually all regulatory regions of a gene, as well as the *cis*-elements that define expression domains, such as scaffold/matrix attachment regions, and isolate the gene from distal regulation (1). Therefore, BACs are used as an economical surrogate to mouse gene-targeting technology, i.e., to *knock-in* large fragments of human genomic DNA for the generation of humanized mice (1). Humanized mice allow stable mouse lines to be established for the investigation of individual human genes and hence for the expression and study of immunological effectors involved in human immune regulation, pathologies, and diseases. In the present study we established a BAC transgenic mouse model, to determine the role of the human CD83 protein. CD83 is a member of the immunoglobulin (Ig)G superfamily, conserved amongst species and exists in two isoforms: a membrane bound (mCD83) and a soluble (sCD83) form, the latter one being generated by proteolytic cleavage of the extracellular domain of mCD83 (2). However, both forms are derived from the same transcript (3). mCD83 is a highly glycosylated surface protein of 40–45 kDa and contains 3 domains: an extracellular Ig-like V domain at the N terminus, a short intracellular cytoplasmic domain of 39aa, and one transmembrane domain (4). As demonstrated recently by a CD83 *knock-in* reporter mouse, the murine CD83 promoter is differently active in various cell types of the immune system. Whereas, strong murine CD83 promoter activity could be detected during the differentiation of dendritic cells (DCs) and B cells, only weak or very weak promoter activity was found in naïve CD4^+^ and CD8^+^ peripheral T cells, respectively (5). Moreover, murine as well as human regulatory T cells (Tregs) were reported to express CD83 and to be essential for Treg cell differentiation and stability (6), thereby defining CD83 as a new lineage marker for T cells with a regulatory phenotype in mice *in vitro* and *in vivo* (7).

Although recently the TLR4/MD-2 complex on CD14^+^ monocytes has been identified as the ligand for sCD83 (8), the precise mode of action of mCD83 and sCD83 is not fully understood yet. Analyses of complete CD83 knock-out mice reported thymic CD83 expression to be essential for the maturation of double positive thymocytes into CD4^+^ T cells (9). This was supported by a more recent publication showing that CD83 on thymic epithelial cells (TECs) is crucial for CD4^+^ T cell selection, thereby reflecting its capacity to attenuate MHC II turnover in cortical TECs by counteracting March8-mediated MHC II ubiquitination (10). Moreover, viruses such as human cytomegalovirus and herpes simplex virus 1 have been described to induce down-modulation of CD83 on human DCs, followed by specific immune evasion strategies, which lead to suppressed antiviral immune responses (11–13). Knock-down of CD83 in human DCs by RNA interference on the other hand led to a decreased capacity of DCs to stimulate T cells in an allogeneic mixed lymphocyte reaction which was accompanied by changes in cytokine expression during T cell priming (14). Generation of a B cell—specific CD83 conditional knock-out (CD83 B-cKO) revealed that those B cells were defective in up-regulating MHC class II and CD86 expression and showed an impaired proliferative capacity after treatment with different stimuli (15). Furthermore, CD83 B-cKO mice had increased numbers of dark zone B cells after immunization with specific antigens, and elicited enhanced IgE responses, indicating that CD83 in B cells is involved in cell activation, germinal center composition and IgE antibody responses (15). Soluble CD83 has been reported to possess potent immune-modulatory properties in different murine autoimmune and transplantation models. Using a cornea transplantation model recombinant sCD83 prolonged allogeneic graft survival via the induction of Tregs and upregulation of the immune-regulatory enzymes IDO as well as TGF-β (16). In addition, sCD83 has been demonstrated to prevent graft rejection of heart, kidney and skin transplants (17, 18). Under inflammatory conditions, typical e.g., for inflammatory bowel disease, the administration of sCD83 not only ameliorated clinical disease symptoms, but also drastically reduced mortality, and strongly decreased inflammatory cytokine expression in mesenteric lymph nodes and colon (19). Moreover, sCD83 showed profound immune-modulatory effects on the induction of autoantibodies in a SLE mouse model and prevented the onset of experimental autoimmune encephalomyelitis (EAE) in the mouse model for multiple sclerosis (20, 21).

As the precise mode of action of CD83 is not completely understood, we decided to study the function of human CD83 in more detail in a physiological context using a humanized CD83-BAC-transgenic mouse model. Therefore, human CD83 *knock-in* transgenic mice were generated resulting in two different founders, BAC-hCD83-2^tg^ and BAC-hCD83-77^tg^. These mice showed a normal composition of different immune cell types and a physiological expression of murine and human CD83 on DCs, B cells, and distinct T cell populations. Interestingly, induction of EAE in these BAC-hCD83 mice resulted in a significant amelioration of disease symptoms and an increased frequency of Tregs in the CNS in comparison to wild-type littermate mice. Furthermore, BAC-hCD83 derived Tregs showed a clearly enhanced activation and suppressive activity. These data strongly support the importance of CD83 for the regulatory phenotype of Tregs *in vitro* as well as *in vivo*.

## Materials and Methods

### Transgenic Mice

Vector pBAC RP11-93F11 was obtained from the Children's Hospital Oakland Research Institute, Oakland, CA 94609 and linearized by MluI digestion controlled by pulsed-field gel electrophoresis. Pronuclear injection was performed by R. Naumann at the Max Planck Institute of Molecular Cell Biology and Genetics, Dresden, Germany. The BACs (1–2 ng/ml) were injected with into the pronucleus of C57BL/6JOlaHsd fertilized oocytes (22). About 2 h after injections, the surviving embryos were transferred into Crl:CD1(ICR) pseudopregnant recipient female mice (20–25 embryos per recipient). Genotyping of obtained transgenic mice was done by PCR of purified tail genomic DNA under standard conditions with primers amplifying short regions of the (i) 5′end of the CD83 non-translated region (P1, for 5′-gcgcagagctcgggactaac-3′, rev 5′-gctcaataaaggtagtatagacc-3′), (ii) 5′ end of exon 1 (P2, for 5′-catttttagtaaagaccaggttcacca-3′, rev 5′-tatcctttggccttttccatgag-3′), (iii) intron 4 (P3, for 5′-aatgacaaggggttctatgc-3′, rev 5′-ggacacagtgagtggcaag-3′), (iv) 3′ end of exon 5 (P4, for 5′-GTAGTGAGATAGCATTGTGAAC-3′, rev 5′-TTTGGATTCTGATTCTCAAACTGG-3′), and (v) the 3′end of the CD83 non-translated region (P5, for 5′-gcgcagagctcgggactaac-3′, rev 5′-gctcaataaaggtagtatagacc-3′). Founder mice were bred at the central animal facility PETZ of the FAU Erlangen.

### BMDC Cultures

Bone marrow derived dendritic cells (BMDCs) from WT^tg^, BAC-hCD83-2^tg^, and BAC-hCD83-77^tg^ were generated from precursor cells as described before (23). In brief: 2 × 10^6^ BMDCs per 10-cm dish (BD Falcon) were cultured for 8 days in R10 medium consisting of RPMI1640 (Lonza), 1% Penicillin/Streptomycin/L-Glutamine (Sigma-Aldrich, Germany), 2-ME (50 μM, Sigma-Aldrich), 10% heat-inactivated FBS (Fetal Bovine Serum Gold, GE Healthcare) and additionally supplemented with GM-CSF supernatant (1:10) from a cell line stably transfected with the murine GM-CSF (24). At days 3 and 6, 10 ml of fresh R10 supplemented with GM-CSF supernatant (1:10) was added, with removing 50% of the old cell culture supernatant at day 6 before. Maturation of BMDCs was induced at day 8 by the addition of 0.1 ng/ml LPS (Sigma-Aldrich) or 500 U/ml TNF-α (Peprotech) for 20 h. At day 9, cells were used for further experiments.

### Generation of Human Monocyte-Derived Dendritic Cells

Human monocyte-derived dendritic cells (DCs) were generated as previously described (25). In brief, peripheral blood mononuclear cells (PBMCs) were prepared from leukoreduction system chambers (LRSCs) of healthy donors by density centrifugation, followed by plastic adherence on tissue culture dishes (BD Falcon). The non-adherent cell fraction was removed and the adherent cell fraction was cultured for 4 days in DC-medium consisting of RPMI 1640 (Lonza) supplemented with 1% (vol/vol) of heat-inactivated human serum type AB (Sigma-Aldrich), 1% Penicillin/Streptomycin/L-Glutamine (Sigma-Aldrich) and 10 mM Hepes (Lonza) as well as 800 IU/ml (day 0) or 400 IU/ml (day 3) recombinant human granulocyte macrophage colony-stimulating factor (GM-CSF) and 250 IU/ml (day 0 and 3) recombinant IL-4 (both Miltenyi Biotec). On day 4, maturation of DCs was induced by adding 0.1 ng/μl LPS (Sigma-Aldrich) for 20 h.

### T- and B Cell Cultures

CD8^+^, CD4^+^CD25^−^, CD4^+^CD25^+^, and B cells were isolated from whole spleen cells by means of MACS using the mouse CD8^+^-, or CD4^+^CD25^+^ Regulatory T cell Isolation Kit, or CD19 Microbeads (all Miltenyi Biotec), according to the manufacturer‘s instructions. Afterwards, 1 × 10^6^ sorted T- and B cells per ml in R10 medium were seeded into 24-well plates and were either left untreated or stimulated with Dynabeads Mouse T-Activator CD3/CD28, according to the manufacturer's instructions (Invitrogen/Thermo Fisher Scientific), or with 50 ng/ml PMA plus 500 ng/ml Ionomycin (both Sigma-Aldrich), or 5 μg/ml LPS (Sigma-Aldrich) for 5–20 h, respectively. Purity of sorted cells was assessed by FACS directly after isolation and revealed ≥92%.

### Analyses of Spleen and Lymph Node Cells

Spleens from mice were isolated, single cell suspensions were prepared and incubated with ammonium chloride solution (1.6 % [v/v] in ddH_2_O; Carl Roth, Karlsruhe, Germany) at 37°C for 5 min to lyse the erythrocytes. Splenocytes including spleen-derived endogenous DCs were then either subsequently analyzed by FACS, or maintained in R10 medium (5 × 10^6^ cells per well in a 24-well plate) in the presence of 0.1 ng/ml LPS (Sigma-Aldrich) or 500 U/ml TNF-α (Peprotech) for 16 h before FACS analyses were performed. For qRT experiments, 2 × 10^6^/ml spleen- or lymph node cells were seeded in R10 medium in 24 well plates and stimulated with 50 ng/ml PMA plus 500 ng/ml Ionomycin (both Sigma-Aldrich) for 5 h before mRNA was isolated.

### Cytospin Preparation and Immunostaining

Cytospin samples from murine WT^tg^, BAC-hCD83-2^tg^, and BAC-hCD83-77^tg^ BMDCs were made by cytocentrifugation (Cytospin3; Shandon; Medite GmbH) of 200 μL fluid (2 × 10^4^ cells) at 1,000 rpm for 10 min on a Superfrost plus slide (Thermo Scientific, Braunschweig, Germany). Samples from human DCs were prepared as controls. Cytospins were air dried overnight and fixed in aceton for 3 min at −20°C. Immunohistochemistry was performed by using an avidin-biotin peroxidase detection system (Vector ABC Elite Kit, Vector laboratories, Burlingame, CA) according to manufacturer's protocol. Endogenous peroxidase activity was inhibited with 3% H_2_O_2_ and blocked with Avidin/Biotin Blocking Kit (Dako, Glostrup, Denmark) before addition of biotinylated anti-hCD83 (clone HB15e, Biolegend). Sections were counterstained with Mayer Hämalaun and mounted in Aquatex (Merck, Darmstadt, Germany). Images were observed using the light microscope Nikon Eclipse Ci.

### Mixed Lymphocyte Reaction (MLR)

At day 9, titrated numbers of immature and matured BMDCs, either derived from BALB/c or transgenic BAC and -WT mice, were co-cultured for 72 h in 96-well flat bottom plates (BD Falcon) with 2 × 10^6^ T cells derived from transgenic BAC, -WT, or BALB/c lymph node cells, respectively. Cell cultures were then pulsed with 1 μCi/well (PerkinElmer) for 8–16 h before they were harvested onto glassfiber filtermates using an ICH-110 harvester (Inotech). Filters were counted in a 1450 microplate counter (Wallac). Additionally, aliquots of cell culture supernatants were cryopreserved for cytokine assays before pulsing with[^3^H]-thymidine when indicated.

### Suppression Assay

For the suppression assay, T-effector (Teff), and Tregs were co-cultured on feeder cells. These feeder cells derived from spleenocytes of RAG1^−/−^ mice, which were treated with Low-Tox®-M Rabbit Complement (Cedarlane) and an anti-mouse CD90.2 antibody (30-H12; BioLegend). Than 5 × 10^4^ cells feeder cells were seeded into 96-well roundbottom plates (BD Falcon). CD4^+^CD25^−^ Teff and CD4^+^CD25^+^ Tregs were isolated from transgenic BAC and –WT mice using the mouse CD4^+^CD25^+^ Regulatory T cell Isolation Kit (Miltenyi Biotec), according to the manufacturer‘s instructions. Purity of sorted cells was assessed by FACS directly after isolation and revealed a purity of ≥92%. Teff were labeled with 0.75 μM Cell Trace Violet (CTV; Life Technologies) per 10^6^ cells and 5 × 10^4^ CTV^+^ Teff cells per well, were added to the RAG1^−/−^ feeder cells. Finally, Tregs were plated onto these cells in titrated numbers and co-cultures were stimulated using 0.25 mg/ml of an anti-mouse CD3ε antibody (145-2C11; BioLegend) and incubated for 96 h before percentage of proliferating cells was determined by FACS. Results were analyzed using FCS Express Flow Cytometry Software (De Novo software). Additionally, aliquots of cell culture supernatants were cryopreserved for cytokine assays.

### Flow Cytometric Analyses (FACS)

For cell surface staining, cells were stained with specific monoclonal antibodies (mAb) for 30 min at 4°C in Dulbecco's PBS (Lonza), washed once and finally resuspended in PBS containing 0.1 μg/ml propidium iodide (PI; Carl Roth) or LIFE/DEAD® Aqua fluorescent dye (Thermo Fisher Scientific). Intracellular Foxp3 and CD83 staining was performed according to the manufacturer‘s instructions (anti-mouse Foxp3-PE [FJK-16S; eBioscience)], CD83-PE (Michel-19) anti-human CD83-PE (HB15e), Foxp3 staining set [eBioscience]). For intracellular staining of CNS infiltrating immune cells, cells were fixed, permeabilized, and intracellular staining was performed in Permeabilization Reagent (Thermo Fisher, Cat. 00-5523-00). Stained cells were immediately analyzed with a FACScan or FACSCanto II cell sorter (BD Biosciences). Cell debris and dead cells were excluded from the analyses by gating on proper forward and sideward light scatter and on PI or LIFE/DEAD® negative cells. A minimum of 10^4^−10^5^ living cells was used for each sample and results were analyzed using FCS Express Flow Cytometry Software (De Novo software). The following mAb obtained from BD Bioscience, eBioscience, or BioLegend were used: Anti-mouse CD3-PerCP/-BV421 (145-2C11), CD4-PerCp/-FITC/-APC Fire (RM4-5), CD8-PerCp/-FITC/-BV510 (53-6.7), CD11b-PerCpCy5.5 (M1/70)/-PECy7 (M1/70), CD11c-PerCp/-BV421 (N418)/-APCFire (N418), CD19-FITC (1D3), CD25-PerCPCy5.5 (PC61), CD45-FITC (B12.3), CD69-PE (H1.2F3), CD80-FITC (16-10A1), CD83-PE/-FITC (Michel-19), CD25-FITC (PC61; 1D3), B220-PE (RA3-6B2), NK1.1 PE (PK136), and anti-human CD83-FITC/-APC (HB15e),CX3Cr1-PECy7 (Sa011F11), FoxP3-eF405 (FJK-16s), GM-CSF-PE (MP1-22E9), I-A/I-E-BV510 (M5/114.15.2)/-BV421 (M5/114.15.2),IL-17A+F –AF647 (TC11-18H10.1), IFN-γ-PECy7 (XMG1.2), Ly6C-APCCy7 (HK1.4), Ly6G-PE (1A8), SiglecH-PerCPCy5.5 (551).

### Cytokine Assay

To determine cytokines secreted into the cell culture supernatants, the LEGENDplex^TM^ Mouse Th Cytokine Panel (13-plex) from BioLegend was used as specified by the manufacturer.

### BCA Protein Assay

Protein concentrations of cell lysates for SDS-PAGE were determined by PierceTM Protein Assay Kit (Thermo Fisher Scientific), according to the manufacturer's protocol.

### SDS-PAGE and Western Blotting

Whole cell extracts for SDS-PAGE were generated as follows (26): 1 × 10^6^ BMDCs or B cells were washed with ice-cold PBS and resuspended in 50 μl lysis buffer mixed with Na_3_VO_4_, NaF, and PMSF. Afterwards, protein concentrations were determined by the BCA protein assay described above. Sample proteins were boiled in the presence of a loading dye mixed with SDS and 2-ME for 10 min at 95°C. Then, 20 μg of total protein per sample were separated on a 12.5% polyacrylamide gel and afterwards transferred onto nitrocellulose filters (Schleicher & Schüll/GE Healthcare), with a pore size of 0.2 μm with the wet blotting device “Mini-Protean II Cell and System” (BioRad). Membranes were incubated with primary antibodies against human CD83 (1G11, kindly provided by Elisabeth Kremmer, LMU Munich, Germany), murine CD83 (R&D Systems) or Beta-actin (AC-74; Sigma-Aldrich), followed by HRP-conjugated goat anti-rat IgG (Cell Signaling Technology), donkey anti-goat IgG (Promega), or goat anti-mouse IgG (Promega) antibodies. Detection was performed with the chemiluminescent ECL Prime Western Blotting Detection Reagent (Amersham, GE Healthcare) on a high performance chemiluminescence film (GE Healthcare).

### Quantitative Real-Time PCR (qRT)

To prepare total RNA from single cell suspensions of BMDCs, T cells, B cells, splenocytes or lymph node cells, either the RNeasy Plus Mini Kit or the RNeasy Plus Micro Kit (Qiagen; both with removal of genomic DNA contamination) were used, as specified by the manufacturer. Pieces of organ tissues derived from perfused mice were homogenized using innuSPEED Lysis Tubes P or I and a SpeedMill Plus (all Analytik Jena) before isolation of total RNA with the RNeasy Plus Mini/Micro Kit (Qiagen). Purity and concentration of RNA was determined by a NanoDrop 2000c Spectrophotometer (PeqLab/ VWR). Subsequently, 1 μg RNA was reverse transcribed into sscDNA using the First Strand cDNA Synthesis Kit (Fermentas), according to the manufacturer‘s protocol. Quantitative Real-Time PCR (qRT) was performed using the CFX96 Real-Time PCR Detection System (BioRad), iQ SYBR Green Supermix (BioRad), and specific primers as stated in Table 1 according to the MIQE guidelines (27). The levels of gene expression normalized to the corresponding house keeper gene were calculated with the comparative Ct (threshold cycle) method, where the data were expressed as 2^−ΔΔ*CT*^.

**Table 1:** Primer list for quantitative real-time PCR.

**Primer**	**Sequence 5^**′**^ ->3^**′**^**
huCD83_for	TGCTGCTGGCTCTGGTTATT
huCD83_rev	TGTGAGGAGTCACTAGCCCT
CD83_for	CGCAGCTCTCCTATGCAGTG
CD83_rev	GTGTTTTGGATCGTCAGGGAATA
HPRT_for	GTTGGATACAGGCCAGACTTTGTTG
HPRT_rev	GATTCAACTTGCGCTCATCTTAGGC
Rpl4_for	GCTGAACCCTTACGCCAAGA
Rpl4_rev	TCTCGGATTTGGTTGCCAGT
Foxp3_for	CCCAGGAAAGACAGCAACCTT
Foxp3_rev	CCTTGCCTTTCTCATCCAGGA
IFNγ_for	GCTTTGCAGCTCTTCCTCAT
IFNγ_rev	GTCACCATCCTTTTGCCAGT
Tbet_for	AGCAAGGACGGCGAATGTT
Tbet_rev	GGGTGGACATATAAGCGGTTC
CTLA-4_for	CCAGAACCATGCCCGGATT
CTLA-4_for	CTGTTGGGGGCATTTTCACA
IL-10_for	CCAAGCCTTATCGGAAATGA
IL-10_rev	TTTTCACAGGGGAGAAATCG

### EAE Induction

EAE was induced as described before (20). In brief: mice aged 10–12 weeks were immunized s.c. with 50 μg myelin oligodendrocyte glycoprotein (MOG)_35−55_ peptide (Charitè Berlin) emulsified in IFA, which was enriched with 10 μg/ml Mycobacterium tuberculosis (H37Ra; Difco/BD). Additionally, 200 ng pertussis toxin (List/Quadratec) was injected i.p. at days 0 and 2 of EAE induction. Scoring of EAE symptoms was performed as follows: 0, no disease; 1, tail weakness; 2, paraparesis; 3, paraplegia; 4, paraplegia with forelimb weakness; and 5, moribund or dead animals.

### Neuropathology

For the visualization of inflammatory infiltrates, spinal cords from perfused EAE mice were harvested on day 17, fixed in liquid nitrogen and stored at −80°C. Preparation of spinal cord sections and Luxol fast blue-staining to indicate demyelinated areas was performed according to protocols as described previously (28).

### Preparation and Restimulation of CNS Infiltrating Cells

Brain and spinal cord derived from perfused mice were homogenized using a Douncer Homogenizer (Kimble, USA). Afterwards, myelin was removed from the cell suspension by a one-step density gradient centrifugation (29) using 37% Percoll (Sigma-Aldrich). Harvested cells were washed in 1x HBSS (Life Technologies, USA) and resuspended in RPMI1640 without phenol red (Life Technologies) for culture or were used directly for flow cytometry. For intracellular staining of cytokines, immune cells derived from CNS were restimulated with 50 ng/ml PMA and 500 μg/ml ionomycin (Sigma) for 5 h with Golgi-transport inhibitors (Golgi-Plug and Golgi-Stop, BD) added for the last 4 h.

### Isolation and Activation of Splenocytes Derived From EAE Mice

On day 17 after EAE induction, the spleen was removed and single cell suspensions were prepared. Spleen cells were incubated with ammonium chloride solution (1.6 % [v/v] in ddH_2_O; Carl Roth, Karlsruhe, Germany) at 37°C for 5 min to lyse the erythrocytes. Subsequently, cells were used for further analyses or maintained in culture medium overnight prior to stimulation with phorbol 12-myristate 13-acetate (PMA; 50 ng/ml), ionomycin calcium salt (ionomycin; 500 ng/ml), GolgiPlug (1 μl/ml), and GolgiStop (0.6 μl/ml; all from Sigma-Aldrich) for 6 h.

### Restimulation Assay

4 × 10^5^ cells derived from spleen of EAE mice were seeded in 96-well cell culture plates (BD Bioscience) in 200 μl of RPMI 1640 medium (Lonza, Basle, Switzerland) supplemented with 10 % (v/v) FCS (PAA/GE Healthcare, Little Chalfont, UK), 1 % (v/v) penicillin/streptomycin/L-glutamine (PAA/GE Healthcare), 50 μM 2-mercaptoethanol (Sigma-Aldrich), and 10 % (v/v) GM-CSF supernatant derived from a cell line transfected to express murine GM-CSF. Subsequently, splenocytes were restimulated with different concentrations of MOG35-55 peptide as indicated. After 3 days, cells were pulsed with 3H-thymidine (1 μC/well; PerkinElmer, Rodgau, Germany) for 16 hours. The culture supernatants were harvested onto glass fiber filtermates using an ICH-110 harvester (Inotech Bioscience, Rockville, Maryland, USA) and filters were counted in a Wallac 1420 Victor2 Microplate Reader (PerkinElmer).

### Statistical Analyses

Statistical calculations were performed using GraphPad Prism software as indicated for each individual experiment. *p* < 0.05 was considered significant.

### Approvals and Legal Requirements for the Project

For the generation of moDCs from leukoreduction system chambers of healthy donors the positive vote from the local ethics committee has been obtained (Re.-Nr.: 4556). All animal experiments were performed in accordance with the European Communities Council Directive (86/609/EEC), and were approved by the local ethics committee (Government of Middle Franconia, Germany).

## Results

### Cellular Composition of Splenocytes and Dendritic Cell Activation Is Unaltered in BAC-hCD83 Transgenic Mice

**Figure 1:**
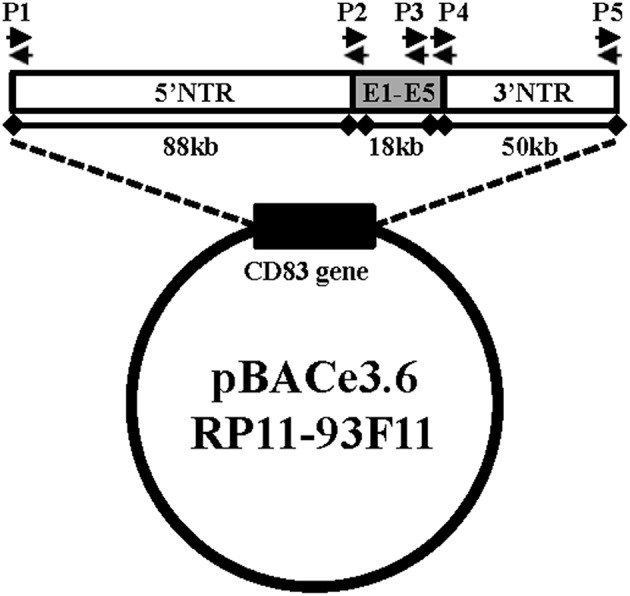
Generation of BAC-hCD83^tg^ mice. pBACe3.6 RP11-39F11 was linearized by *MluI* digestion before microinjection into the pronuclei of fertilized mouse eggs on a C57BL/6 background. The vector contains 156 kb of the human CD83 gene locus including ~88 kb of the 5′ non-translated region (NTR), the 18 kb coding region including Exons 1–5 (E1–E5) and ~50 kb of the 3′ NTR. Primer pairs P1–P5 used for screening of founder mice and littermates are indicated by black arrows.

BAC-hCD83 transgenic mice were generated by pronuclear injection of the linearized vector pBAC RP11-93F11 (Figure 1) and gave finally rise to two heterozygous founder mice, i.e., BAC-hCD83-2^tg^ and BAC-hCD83-77^tg^. As determined by PCR (Figure 1), the BAC-hCD83-2^tg^ founder inserted the whole 156 kb long human sequence into the mouse genome, whereas the BAC-hCD83-77^tg^ founder inserted only the 18 kb long CD83 coding region (data not shown).

**Figure 2:**
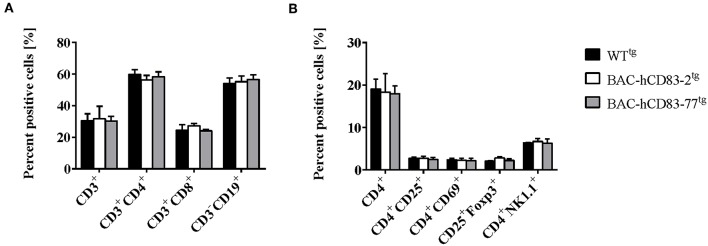
Cellular composition of splenocytes is unaltered in BAC-hCD83^tg^ mice. Spleens were removed from WT^tg^, BAC-hCD83-2^tg^ and BAC-hCD83-77^tg^ mice and isolated cells were immediately analyzed by flow cytometry (FACS). Splenocytes were either stained with antibodies specific for T- (CD3, CD4, CD8) and B cell (CD19) markers **(A)** or activation markers for CD4^+^ T cells (CD25, CD69), for regulatory T cells (CD25, Foxp3), or NK cells (CD4, NK1.1) **(B)**. Percentages are presented as mean + SEM, *n* = 4. Data were analyzed using the two-way ANOVA with Tukey's test. Bars without annotation are not significant (*P* ≥ 0.05).

We first analyzed the cellular composition of naïve spleen cells by flow cytometric analyses. As shown in Figure 2A, there were no differences in the absolute numbers of CD3^+^ T cells, CD4^+^, and CD8^+^ T cells or CD19^+^ B cells in comparison to splenocytes derived from negatively screened WT^tg^ mice. Moreover, there was no differential distribution of activated CD4^+^CD25^+^, CD4^+^CD69^+^ cells, or of CD25^+^Foxp3^+^ regulatory T cells (Tregs) or CD4^+^NK1.1^+^ natural killer cells (Figure 2B).

**Figure 3:**
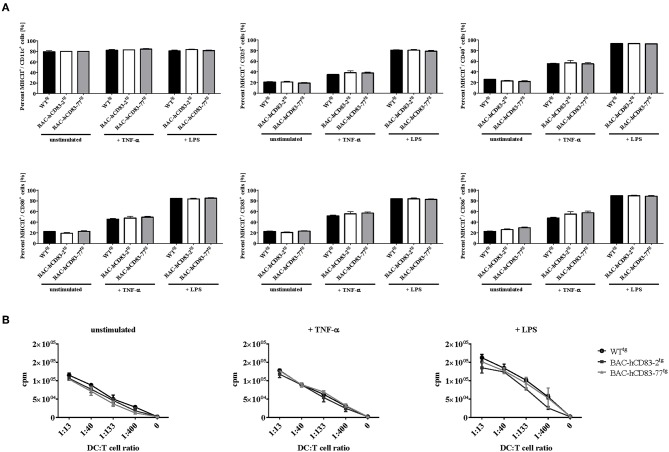
Maturation status and function of BMDCs derived from BAC-hCD83^tg^ mice are comparable to WT^tg^ derived cells. BMDCs were generated in the presence of GM-CSF from precursor cells and were either left unstimulated, or matured at day 8 using 0.1 ng/ml LPS or 500 U/ml TNF-α for 20 h. **(A)** Flow cytometric analyses of the cell surface molecules CD11c, MHC class II, CD25, CD80, CD83, and CD86 on immature(“unstimulated”), TNF-α matured, or LPS-matured BMDCs. Percentages are presented as mean + SEM, *n* = 3. Data were analyzed using the two-way ANOVA with Tukey's test. Bars without annotation are not significant (*P* ≥ 0.05) in comparison to the respective WT^tg^ control. **(B)** Allogeneic mixed lymphocyte reaction (MLR) of titrated numbers of immature, TNF-α, or LPS-matured BMDCs, and T cells derived from BALB/c lymph nodes. Counts per minute (cpm) are presented as mean + SEM, *n* = 3.

Next, bone-marrow derived dendritic cells (BMDCs) were generated from BAC-hCD83^tg^ and WT^tg^ mice and were either left unstimulated or stimulated with TNF-α or LPS for 20 h. These cells were then phenotypically analyzed by FACS and their functionally was characterized using an allogeneic mixed lymphocyte reaction (MLR). FACS analyses (Figure 3A) revealed no differences in the expression of CD86, CD83, CD80, CD25 on CD11c^+^ MHCII^+^ BMDCs, regardless if the cells were immature, TNF or LPS matured. This was also functionally reflected by the MLR analyses (Figure 3B), where no significant differences were observed between WT^tg^ and BAC-hCD83^tg^ derived BMDCs. Generally, forced expression of human CD83 did not alter survival, phenotype, behavior, or breeding of BAC-hCD83-2^tg^ and BAC-hCD83-77^tg^ mice. Taken together, BAC-hCD83^tg^ and WT^tg^ mice are comparable regarding cell type distribution in the spleen and activation status and functionality of BMDCs.

### Expression of huCD83 in Tissues and by Immune Cells

**Figure 4:**
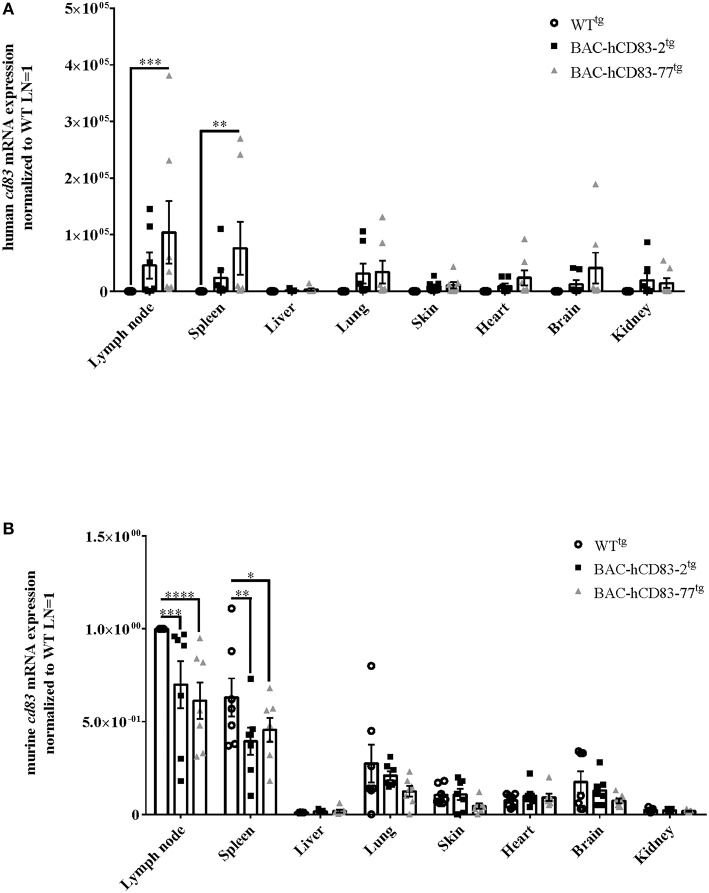
Analyses of human and murine CD83 expression in various organs. RNA was prepared from lypmhe node, spleen, liver, lung, skin, heart, brain, and kidney of perfused WT^tg^, BAC-hCD83-2^tg^, or BAC-hCD83-77^tg^ mice and immediately transcribed into cDNA. Afterwards, quantitative Real-time PCR was performed regarding human CD83 **(A)** or murine CD83 **(B)** and HPRT as control. Gene expression levels were normalized to the corresponding *hprt* values and calculated with the comparative Ct method, whereby data are expressed as 2^−ΔΔ*CT*^. Resulting data were finally normalized to values derived from WT^tg^ lymph node cells = 1. Data shown are presented as mean + SEM, *n* = 7. Data were analyzed using the two-way ANOVA with Dunnett's test; ^*^P < 0.05, ^**^P < 0.01, ^***^P < 0.001, ^****^P < 0.0001 bars without annotation are not significant (*P* ≥ 0.05).

To evaluate the expression of human vs. murine CD83 in different organs and cells, lymph nodes, spleen, liver, lung, skin, heart, brain, and kidney were isolated from perfused WT^tg^ and BAC-hCD83^tg^ mice. After RNA isolation and reverse transcription into cDNA, quantitative real-time PCR (qRT) was performed regarding human *CD83* (Figure 4A) and murine *cd83* transcripts (Figure 4B). Notably, highest human and murine CD83 expression was found in lymph nodes and spleen, while CD83 transcripts were almost absent in the liver. Additionally, BAC-hCD83-77^tg^ mice showed a higher expression of human CD83 than BAC-hCD83-2^tg^ mice, which was accompanied by a decreased murine CD83 expression in comparison to WT^tg^ mice. In other organs including lung, skin, heart, brain and kidney, human CD83 expression, of both BAC-hCD83^tg^ founder mice, was only low and murine CD83 almost absent in comparison to WT^tg^ mice.

**Figure 5:**
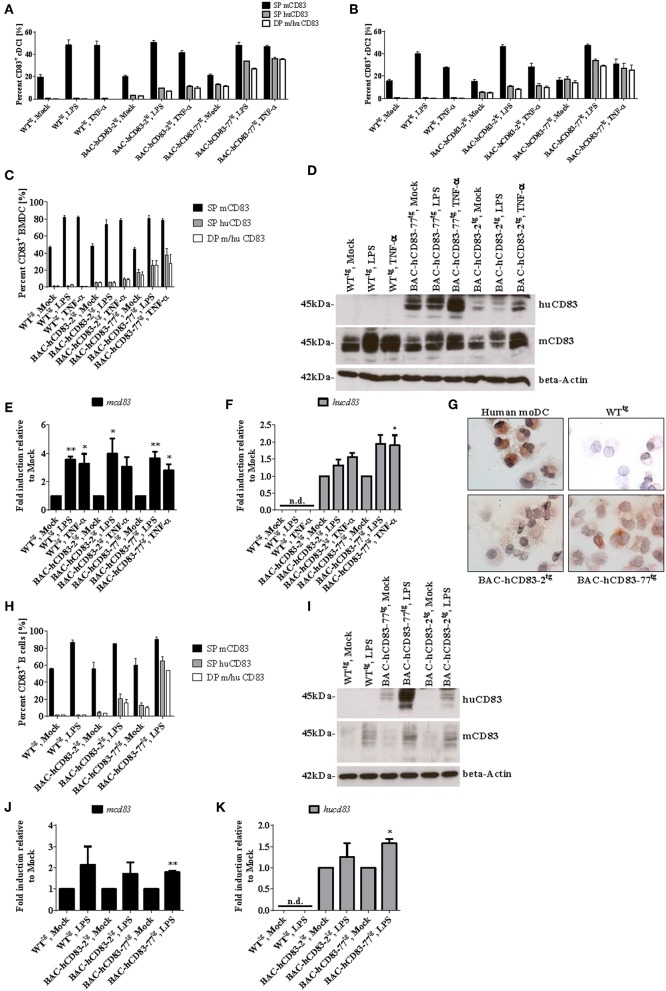
BMDCs and B cells from BAC-hCD83^tg^ mice express human CD83. **(A,B)** Spleen cells from WT^tg^, BAC-hCD83-2^tg^, or BAC-hCD83-77^tg^ mice, either freshly isolated (“Mock”) or stimulated with 0.1 ng/ml LPS, or 500 U/ml TNF-α for 16 h, were analyzed by FACS on the distribution of human (hu) and murine (m) CD83^+^ cDC1 and cDC2 cells. cDC1 cells are defined as MHCII^+^ SiglecH^−^ CD11c^+^ CD11b^int^ CD8^+^, whereas cDC2 are defined as MHCII^+^ SiglecH^−^ CD11c^+^ CD11b^+^ CD8^−^ cells. **(C–G)** BMDCs were generated from WT^tg^, BAC-hCD83-2^tg^, or BAC-hCD83-77^tg^ derived precursor cells and were either left immature or matured at day 8 with 0.1 ng/ml LPS or 500 U/ml TNF-α for 20 h. **(H–K)** B cells were isolated from WT^tg^, BAC-hCD83-2^tg^, or BAC-hCD83-77^tg^ derived whole spleen cells by MACS using CD19 Microbeads. CD19^+^ cells were either left untreated (Mock) or stimulated with 5 μg/ml LPS (Sigma-Aldrich) for 20 h. **(A–C,H)** Flow cytometric analyses regarding the co-expression of human (huCD83) and murine CD83 (mCD83). SP = single positive cDC1 **(A)**, cDC2 **(B)**, BMDCs **(C)** or B cells **(H)**, DP = double positive cells. Percentages are presented as mean + SEM, *n* = 3. **(D,I)** Western Blot analyses of cell lysates of BMDCs **(D)**, or B cells **(I)** on huCD83, mCD83, and beta-Actin protein expression. One representative experiment out of three is shown. **(E,F,J,K)** Quantitative Real-time PCR of cDNA derived from BMDCs **(E,F)** or B cells **(J,K)** on *mcd83* **(E,J)** and *hucd83* **(F,K)**. Gene expression levels were normalized to the corresponding *hprt* values and calculated with the comparative Ct method, whereby data are expressed as 2^−ΔΔ*CT*^. Resulting data were finally normalized to values derived from Mock = 1. Data shown are presented as mean + SEM, *n* = 3. Data were analyzed using one-way ANOVA with Dunnett's test **(E,F)**, or Welch's *t*-test **(J,K)**; ^*^P < 0.05, ^**^P < 0.01, bars without annotation are not significant (*P* ≥ 0.05); n.d. = non-detectable. **(G)** Expression of human CD83 was assessed using an anti-CD83 mAb in combination with cytospin preparations of LPS-matured human monocyte-derived (mo)DCs (upper left panel), WT^tg^- (upper right), BAC-hCD83-2^tg^- (lower left), and BAC-hCD83-77^tg^- (Lower right panel) BMDCs. One representative experiment out of three is shown.

To analyze the human and murine CD83 expression of different cell types in more detail, we first assessed the expression of murine vs. human CD83 on spleen-derived endogenous CD8^+^ DCs (cDCs1) and CD8^−^ DCs (cDCs2), either freshly isolated (“Mock”) or treated with LPS or TNF-α for 16 h. Murine CD83 is expressed on the cell surface of cDC1 and cDC2 cells of WT^tg^ and BAC-hCD83^tg^ mice and is upregulated after stimulation with LPS or TNF-α (Figures 5A,B). Human CD83, however, was only expressed by cDCs1 and cDCs2 derived from huCD83-BAC^tg^ mice after activation of DCs with LPS or TNF-α. hCD83 was slightly upregulated on DCs derived from BAC-hCD83-2^tg^ mice, and highly upregulated on DCs derived from BAC-hCD83-77^tg^ mice (Figures 5A,B). In addition, all cells positive for human CD83 concurrently expressed murine CD83. Next, BMDCs were generated and either left unstimulated or treated with TNF-α or LPS. Subsequent FACS and Western Blot analyses (Figures 5C,D) showed that murine CD83 expression is induced after stimulation and is similar between BMDCs derived from WT^tg^ and BAC-hCD83^tg^ mice. As expected, WT^tg^-derived BMDCs expressed no human CD83, whereas BAC-hCD83^tg^-derived cells did, with the highest expression after TNF-α stimulation. Interestingly, again human CD83 expression was much lower in BAC-hCD83-2^tg^-BMDCs than in BAC-hCD83-77^tg^-BMDCs and only slightly upregulated after stimulation both with LPS and TNF-α. Moreover, all cells which were positive for human CD83, simultaneously expressed murine CD83 (Figure 5C). Corresponding qRT analyses using murine (Figure 5E) and human CD83 specific primers (Figure 5F), confirmed the data observed on protein level in Figures 5C,D. Furthermore, immuno-cytochemical stainings (cytospin preparations), using BMDC confirmed the expression of human CD83 in BAC-hCD83-2^tg^ and BAC-hCD83-77^tg^ (Figure 5G, lower left and right) mice compared to WT^tg^-derived (Figure 5G, upper right) mice. As positive controls human monocyte derived DC were used (Figure 5G, upper left).

Next, CD19^+^ B cells were isolated from spleen and either left unstimulated or stimulated with LPS, followed by FACS-, Western Blot-, and qRT analyses. Murine CD83 was upregulated after LPS-treatment in comparison to mock-treated B cells (Figures 5H–J), whereas human CD83 was not detectable on WT^tg^-derived B cells, either by FACS (Figure 5H), Western Blot (Figure 5I), or on RNA level (Figure 5K). Moreover, no differences in murine CD83 could be detected between cells derived from WT^tg^- or BAC-hCD83^tg^ mice. In contrast, untreated and LPS-stimulated B cells derived from BAC-hCD83-2^tg^ mice and BAC-hCD83-77^tg^ B cells expressed human CD83 (Figures 5H,I,K). Again, B cells derived from BAC-hCD83-2^tg^ mice expressed human CD83 to a much lesser extent than BAC-hCD83-77^tg^ derived B cells (Figures 5H,I). In summary, BMDCs as well as B cells derived from BAC-hCD83^tg^ mice express human CD83 without influencing murine CD83 expression.

**Figure 6:**
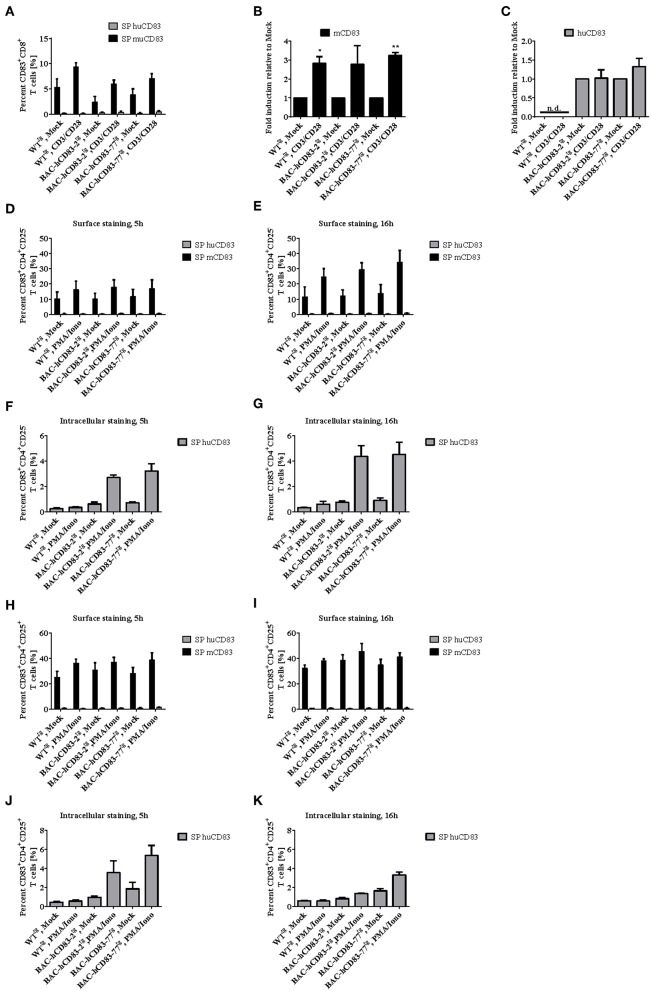
Analyses of CD83 expression on CD8^+^ and CD4^+^ T cell populations. CD8^+^, CD4^+^CD25^−^, and CD4^+^CD25^+^ cells derived from WT^tg^, BAC-hCD83-2^tg^, or BAC-hCD83-77^tg^ mice were isolated from whole spleen cells using MACS technology. T cells were either left unstimulated (mock) or stimulated with Dynabeads Mouse T-Activator CD3/CD28, or with 50 ng/ml PMA plus 500 ng/ml Ionomycin (PMA/Iono), respectively, for 5–20 h. **(A)** Flow cytometric analyses regarding surface expression of human (huCD83) and murine CD83 (mCD83) on CD8^+^ T cells. Percentages are presented as mean + SEM, *n* = 4. **(B,C)** Quantitative Real-time PCR of cDNA derived from CD8^+^ T cells on *mcd83* **(B)** and *hucd83* **(C)**. Gene expression levels were normalized to the corresponding *hprt* values and calculated with the comparative Ct method, whereby data are expressed as 2^−ΔΔ*CT*^. Resulting data were finally normalized to values derived from Mock = 1. Data shown here are presented as mean + SEM, *n* = 3. Data were analyzed with Welch's *t*-test; ^*^P < 0.05, ^**^P < 0.01, bars without annotation are not significant (*P* ≥ 0.05), n.d. = non-detectable. Flow cytometric analyses on surface **(D,E,H,I)** expression of huCD83 and mCD83 as well as intracellular staining on huCD83 **(F,G,J,K)** of CD4^+^CD25^−^ **(D,E,F,G)** and CD4^+^CD25^+^ **(H,I,J,K)** MACS-sorted T cells. SP = single positive cells. Percentages are presented as mean + SEM, *n* = 4.

We next examined the presence of murine and human CD83 molecules on splenic CD8^+^ (Figures 6A–C), CD4^+^CD25^−^ (Figures 6D–G, Supplementary Figure 1) and CD4^+^CD25^+^ (Figures 6G–K, Supplementary Figure 1) T cells. FACS analyses revealed only a very low expression of murine CD83 on CD8^+^ T cells, which was slightly upregulated after stimulation with CD3/CD28 Dynabeads for 20 h on protein- and mRNA level (Figures 6A,B). Human CD83 on the other hand could not be detected on the cell surface of CD8^+^ T cells from BAC-hCD83^tg^ mice (Figure 6A), but was detectable at very low amounts on RNA level (Figure 6C). Expression of human CD83 was slightly increased upon stimulation with CD3/CD28 Dynabeads on BAC-hCD83-77^tg^ derived cells. Regarding murine CD83 specific cell surface staining on CD4^+^CD25^−^ T cells demonstrated a CD83 upregulation after stimulation with PMA/Ionomycin, which was highest at the 16 h time point (Figures 6D,E). Interestingly, in BAC-hCD83^tg^ derived CD4^+^CD25^−^ T cells, human CD83 was only detectable intracellularly and showed highest expression levels 16 h after stimulation (Figures 6D–G, Supplementary Figure 1). These findings are in contrast to the CD4^+^CD25^+^ Treg cells, which slightly upregulate murine CD83 expression on the cell surface after stimulation with PMA/Ionomycin (Figures 6H,I). Human CD83 on the other hand, which again could only be detected intracellularly, showed its highest expression in BAC-hCD83^tg^ derived Tregs 5 h after stimulation, whereas it was downregulated at the later time point (Figures 6J,K, Supplementary Figure 1). Taken together, CD8^+^, CD4^+^CD25^−^, and CD4^+^CD25^+^ T cells derived from BAC-hCD83^tg^ mice express human CD83 after stimulation, but expression levels peak at different time points.

### BAC-hCD83^tg^ Mice Show Ameliorated EAE Symptoms *in vivo* and an Altered T Cell Function *in vitro*

**Figure 7:**
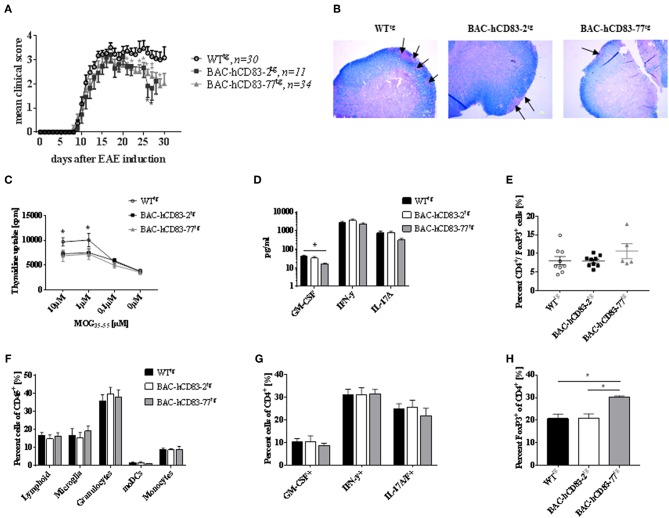
Human CD83 expression in mice lead to reduced EAE symptoms. **(A)** EAE. MOG peptide emulsified in CFA and enriched with M. tuberculosis was injected s.c. to induce EAE in WT^tg^ (*n* = 30), BAC-hCD83-2^tg^ (*n* = 11), and BAC-hCD83-77^tg^ mice (*n* = 34) at day 0. Pertussis toxin was injected i.p. at days 0 and 2 of EAE. Clinical scoring was performed on the indicated time points until day 30. Mean clinical score is presented as mean + SEM. Data were analyzed using the Wilcoxon test. ^*^P < 0.05, ^**^P < 0.01, bars without annotation are not significant (*P* ≥ 0.05). **(B–H)** EAE was induced in in WT^tg^ (*n* = 9), BAC-hCD83-2^tg^ (*n* = 9), and BAC-hCD83-77^tg^ mice (*n* = 5) as described in **(A)**, but mice were sacrificed on day 17 after EAE induction at the peak of disease to analyze the neuropathology **(B)**, the spleen cells **(C–E)**, and the CNS infiltrating immune cells **(F–H)**. **(B)** Luxol fast blue-staining of spinal cord sections harvested from in WT^tg^, BAC-hCD83-2^tg^ and BAC-hCD83-77^tg^ mice at day 17 after EAE induction. Black arrows mark demyelinated areas. Results are representative of at least 3 independent experiments. **(C–E)** Analyses of splenocytes derived from EAE mice. **(C)** MOG-specific proliferation of spleen cells restimulated with 10–0.1 μM MOG_35−55_ peptide for 96 h. Cell proliferation was measured using thymidine incorporation and is shown as cpm ± SEM. **(D)** Cytometric Bead Array (CBA) of cell culture supernatants restimulated with 10 μM MOG_35−55_ peptide. **(E)** Flow cytometric analyses on the frequency of splenic CD4^+^FoxP3^+^ T cells. **(F–H)** Assessment of CNS infiltrating immune cells during EAE by flow cytometry. Total CNS leukocytes (CD45^+^ cells) were classified into total lymphoid cells(CD45^+^CD11b^−^), microglia (CD45^int^CD11b^+^), granulocytes (CD45^+^CD11b^+^Ly6C^int^Ly6G^+^), monocytes (CD45^+^CD11b^+^Ly6C^+^Ly6G^−^), and monocyte-derived DCs (moDCs; CD45^+^CD11b^+^Ly6C^int^Ly6G^int^I-A/I-E^+^CD11c^+^) **(F)**. Intracellular staining of CD45^+^CD4^+^ cells on cytokines GM-CSF, IFN-γ and IL-17A/F **(G)**. Flow cytometric analyses of CD45^+^CD4^+^FoxP3^+^ T cells derived from the CNS **(H)**. Data are presented as mean + SEM and were analyzed using one-way **(D,E,H)** or two-way ANOVA **(C,F,G)** with Tukey's test. ^*^P < 0.05, bars without annotation are not significant (*P* ≥ 0.05).

To investigate the role of endogenous human CD83 expression under inflammatory condition *in vivo*, we used the EAE model, which is an animal model for T-cell-mediated autoimmune diseases. EAE was induced in BAC-hCD83^tg^ and WT^tg^ mice. Upon EAE induction all mice developed classical EAE-associated symptoms, characterized by ascending paralysis 9–11 days after immunization with MOG_35−55_ peptide (Figure 7A). Interestingly, both BAC-hCD83-2^tg^ and BAC-hCD83-77^tg^ mice showed a significant ameliorated disease course from day 20 onwards, when compared to WT^tg^ control mice. To analyze the immunological status of WT^tg^ vs. BAC-hCD83^tg^ mice during EAE in more detail, mice were sacrificed at the peak of disease (day 17) and analyzed. First, Luxol fast blue-staining of spinal cord of EAE mice was performed to visualize the inflammatory infiltrates. As illustrated in Figure 7B, spinal cord of BAC-hCD83-2^tg^ and particularly of BAC-hCD83-77^tg^ mice showed a substantially reduced demyelination in comparison to WT^tg^ mice. Splenic cells from both BAC-hCD83-2^tg^ and BAC-hCD83-77^tg^ mice responded inferior to the antigen-specific restimulation *in vitro* compared to Wt^tg^ mice (Figure 7C). Furthermore, we could not detect an altered cytokine response in those cultures regarding IFN-γ and IL-17, while there was less GM-CSF in the cell culture supernatants containing splenocytes derived from BAC-hCD83-77^tg^ mice (Figure 7D). However, we found a slightly increased number of Foxp3^+^ T cells in the spleen of BAC-hCD83-77^tg^ mice in comparison to WT^tg^ mice (Figure 7E). Next, flow cytometric analyses of infiltrated immune cells of brain and spinal cord at the peak of disease (day 17) were performed, but showed no significant differences in the distribution of total lymphoid cells, microglia, granulocytes, monocyte-derived (mo)DCs, or monocytes (Figure 7F). Likewise, the percentage of GM-CSF-, IFN-γ-, or IL-17 producing CD4^+^ T cells did not deviate, independent of the severity of the clinical symptoms (Figure 7G). However, corresponding to what we observed in the spleen, the number of Foxp3^+^ cells was significantly increased in the CNS of BAC-hCD83-77^tg^ in comparison to WT^tg^ mice (Figure 7H).

**Figure 8:**
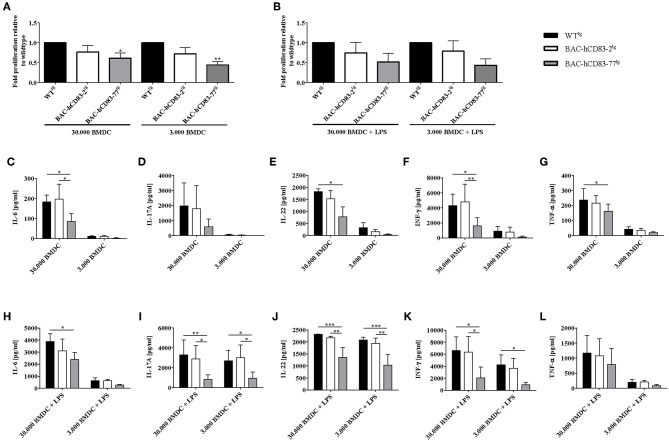
Reduced capacity of BAC-hCD83^tg^ derived LN cells to proliferate and secrete pro-inflammatory cytokines. Titrated numbers of immature (day 9) or LPS-matured BMDCs from BALB/c mice were co-cultured either with cells derived from transgenic BAC, or -WT lymph node cells of healthy non-EAE mice for 72 h. Before pulsing with 1 μCi/well, aliquots of cell culture supernatants were removed. **(A,B)** Mixed lymphocyte reaction comprising immature **(A)** and LPS-matured **(B)** BMDCs. Proliferative capacity of cells is presented as fold induction relative to WT^tg^ as mean +SEM, *n* = 4. Data were analyzed using the one-way ANOVA with Dunnett's test. ^*^P < 0.05, ^**^P < 0.01, bars without annotation are not significant (*P* ≥ 0.05). **(C–L)** Cytokine expression levels (IL-6, IL-17A, IL-22, IFN-γ, TNF-α) present in co-culture derived supernatants with immature **(C–G)**, or LPS-matured **(H–L)** BMDCs, using a Legendplex. Amounts of cytokines [pg/ml] are presented as mean + SEM, *n* = 3. Data were analyzed using two-way ANOVA with Tukey's test. ^*^P < 0.05, ^**^P < 0.01, ^***^P < 0.001, bars without annotation are not significant (*P* ≥ 0.05).

As it has been reported previously that Th1 and Th17 cells mediate pathogenic effects in the EAE model, while Tregs play a regulatory role in the resolution phase of the disease (30), we analyzed the functionality of T cells derived from BAC-hCD83^tg^ mice and compared them with WT^tg^ mice derived cells, in more detail. Therefore, allogeneic mixed lymphocyte reaction (MLR), using titrated numbers of immature or LPS-matured BMDCs derived from BALB/c and lymph node (LN) cells either from healthy non-EAE WT^tg^ or BAC-hCD83^tg^ mice, was performed. After 3 days of co-culture, cell proliferation rates were assessed using a thymidine incorporation assay (Figures 8A,B). In addition cell culture supernatants were removed for cytokine analyses using a cytometric bead array (CBA; Figures 8C–L). The proliferation assays clearly revealed that LN cells derived from BAC-hCD83^tg^ mice responded to a lesser extend to allogeneic immature and mature BMDCs than LN cells derived from WT^tg^ mice (Figures 8A,B). Analyses of corresponding cell culture supernatants showed that co-cultures with immature DCs (Figures 8C–G), contained lower cytokine levels in comparison to co-cultures where mature DCs were present (Figures 8H,I). Furthermore, co-cultures where 30.000 immature DCs and BAC-hCD83-77^tg^ derived LN cells were used generated reduced pro-inflammatory cytokines levels of IL-6, IL-17A, IL-22, IFN-γ, and TNF-α. Similarly, supernatants derived from MLR assays using LPS-matured BMDCs in combination with BAC-hCD83-77^tg^ derived LN cells, showed clearly reduced cytokines levels of IL-6, IL-17A, IL-22, IFN-γ, and TNF-α in comparison to WT^tg^ derived LN cells.

### BAC-hCD83^tg^ Derived Tregs Show a Higher Suppressive Capacity *in vitro* When Compared to WT^tg^-Tregs

**Figure 9:**
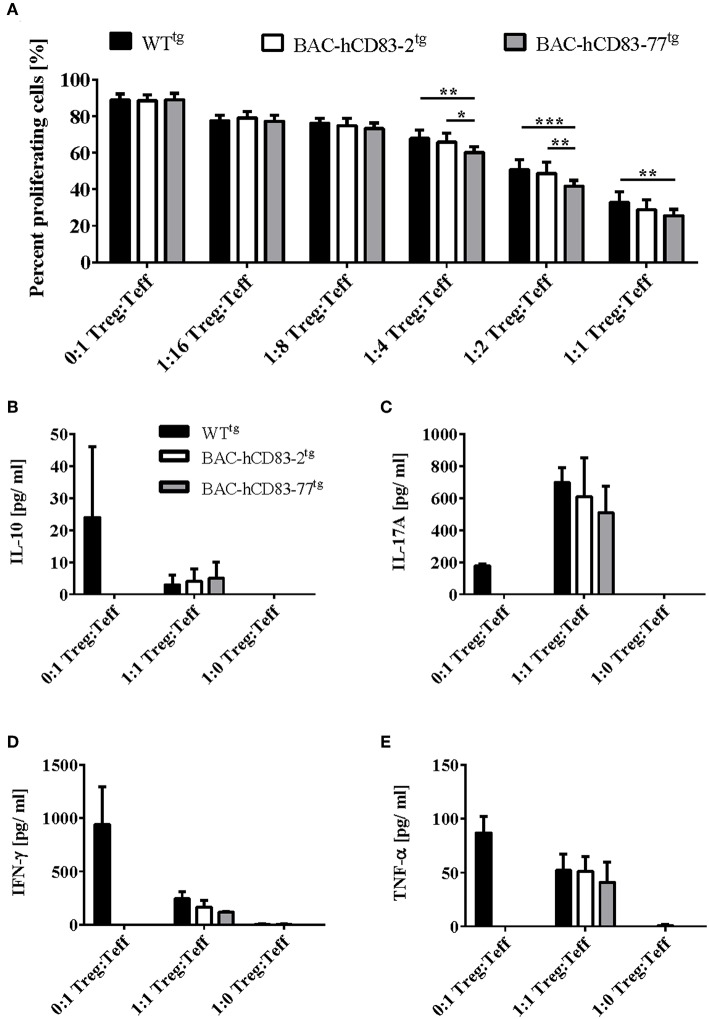
BAC-hCD83^tg^ derived Tregs show a higher suppressive capacity and altered cytokine expression. Titrated numbers of MACS-sorted CD4^+^CD25^+^ regulatory T cells (Tregs) derived from healthy non-EAE BAC- or WT transgenic mice were co-cultured with splenocytes derived from RAG1^−/−^ mice together with Cell Trace Violet (CTV)-labeled WT CD4^+^CD25^−^ T effector (Teff) cells for 96 h. **(A)** Suppressive capacity of Tregs was determined measuring CTV-dilution of Teff by flow cytometry. Percentages are presented as mean + SEM, *n* = 4. **(B–E)** Supernatants derived from Treg—Teff co-cultures were analyzed using a Legendplex for the expression of IL-10 **(B)**, IL-17A **(C)**, IFN-γ **(D)**, and TNF-α **(E)**. Cytokine concentrations [pg/ml] are presented as mean + SEM, *n* = 3. Data shown in **(A–E)** were analyzed using two-way ANOVA with Tukey's test. ^*^P < 0.05, ^**^P < 0.01, ^***^P < 0.001, bars without annotation are not significant (*P* ≥ 0.05).

Since Tregs play a crucial role in the control of EAE symptoms and BAC-hCD83^tg^ derived LN cells show a reduced proliferative capacity in comparison to WT^tg^ LN cells *in vitro*, we next investigated the suppressive capacity of BAC-hCD83^tg^ derived Tregs. Thus, CD4^+^CD25^−^ T-effector (Teff) cells were isolated from C57BL/6 WT mice, labeled with Cell Trace Violet, and co-cultured with titrated numbers of CD4^+^CD25^+^ Tregs derived from healthy WT^tg^ or BAC-hCD83^tg^ mice for 4 days. Afterwards supernatants were removed for cytokine analyses and proliferation of Teff cells was assessed by flow cytometry. Results herein revealed a significantly higher suppressive capacity of BAC-hCD83^tg^ derived Tregs up to a ratio 1:4 Treg:Teff in comparison to Tregs from WT^tg^ mice (Figure 9A). Next, supernatants from the 1:1 Treg:Teff condition were analyzed by CBA showing a trend toward increased IL-10 levels and decreased amounts of pro-inflammatory cytokines including IL-17A, IFN-γ, and TNF-α (Figures 9B–E).

**Figure 10:**
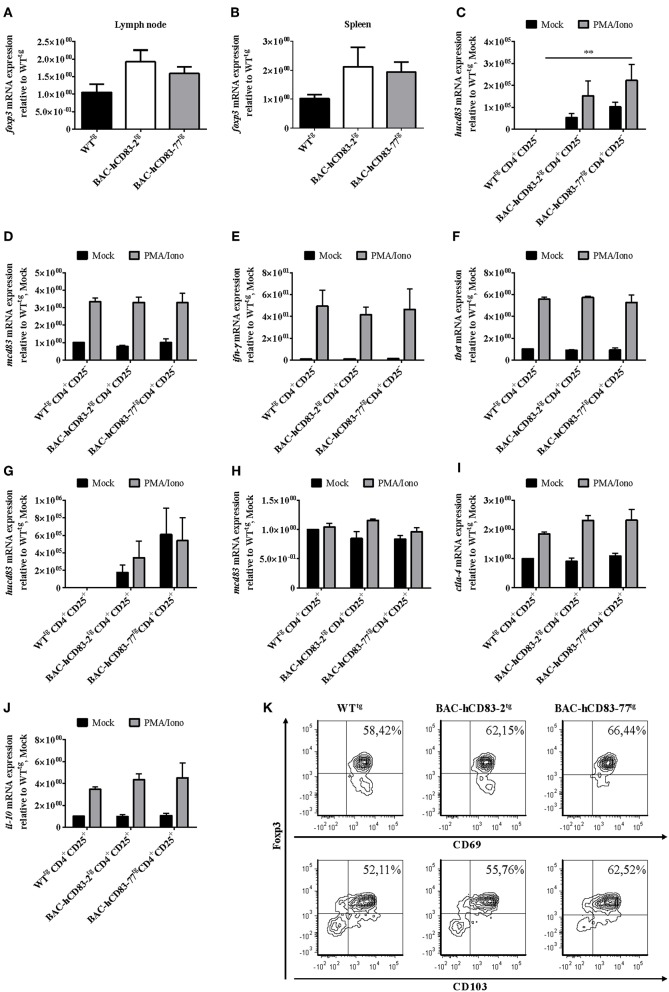
Analyses of activation markers expressed by CD4^+^CD25^−^ and CD4^+^CD25^+^ T cells. **(A,B)** Quantitative Real-time PCR analyses regarding *foxp3* expressed by whole lymph node **(A)** or spleen cells **(B)** of healthy non-EAE WT- or BAC- transgenic mice, stimulated with 50 ng/ml PMA plus 500 ng/ml Ionomycin, for 5 h. Data shown are presented as mean + SEM, *n* = 3. Data were analyzed using the Kruskal-Wallis test; bars without annotation are not significant (*P* ≥ 0.05). **(C–J)** Quantitative Real-time PCR analyses using MACS-sorted CD4^+^CD25^−^ **(C–F)**, or CD4^+^CD25^+^ **(G–J)** cells derived from WT- or BAC- transgenic mice. Cells were either stimulated with 50 ng/ml PMA plus 500 ng/ml Ionomycin (PMA/Iono), for 5 h, or were left unstimulated (Mock). The following transcripts were analyzed: *hucd83* **(C,G)**, *mcd83* **(D,H)**, *ifn-*γ **(E)**, *tbet* **(F)**, *ctla-4* **(I)**, and *il-10* **(J)**. Gene expression levels were normalized to the corresponding *rpl4* values, and calculated using the comparative Ct method, whereby the data are expressed as 2^−ΔΔ*CT*^. Resulting data were finally normalized to values derived from WT^tg^, Mock = 1. Data shown here are presented as mean + SEM, *n* = 3. Data were analyzed using two-way ANOVA with Dunnett's test; ^**^P < 0.01, bars without annotation are not significant (*P* ≥ 0.05). **(K)** Flow cytometric analyses of MACS-sorted CD4^+^CD25^+^ cells stimulated with 50 ng/ml PMA plus 500 ng/ml Ionomycin (PMA/Iono) for 5 h, regarding Foxp3, CD69 (upper panel), and CD103 (lower panel) expression. One representative experiment out of three is shown.

Finally, we isolated whole lymph node (Figure 10A) and spleen (Figure 10B) cells, stimulated them for 5 h with PMA plus Ionomycin and isolated the mRNA, followed by reverse transcription and qRT analyses regarding Foxp3 mRNA expression. A clear increase of Foxp3 expression was observed in lymph node and splenic cells derived from BAC-hCD83^tg^ mice in comparison to WT^tg^ mice. To analyze the activation status of splenic CD4^+^CD25^−^ T conventional cells (T conv, Figures 10C–F) and CD4^+^CD25^+^ (Figures 10G–K) Tregs in more detail, MACS-sorted cells were either left untreated or were stimulated with PMA plus Ionomycin for 5 h. Subsequent qRT analyses demonstrated both Tconv and Tregs derived from BAC-transgenic mice to express human CD83 (Figures 10C,G), while murine CD83 expression (Figures 10D,H) was equal to that of WT^tg^ mice. Moreover, untreated and PMA/Ionomycin stimulated Tconv showed no differences regrading IFN-γ or Tbet expression (Figures 10E,F), while Tregs revealed higher CTLA-4 and IL-10 levels (Figures 10I,J). Additional flow cytometric analyses demonstrated a slightly enhanced expression of the activation markers CD69 and CD103 on stimulated Tregs derived from BAC-hCD83^tg^ mice. A clearly increased number of Foxp3^+^CD69^+^ and Foxp3^+^CD103^+^ cells were observed on cells derived from BAC-hCD83^tg^ mice compared to WT^tg^ animals. Taken together, BAC-hCD83^tg^ derived Tregs showed a higher suppressive capacity when compared with WT^tg^ derived Tregs which was accompanied by an increased expression levels of CTLA-4, IL-10, CD69, and CD103.

## Discussion

Transgenic mice offer an interesting experimental *in vivo* system to analyse the effects of specific gene overexpression. Bacterial artificial chromosome (BAC) vectors, which can accommodate large genomic intervals of up to 300 kb and are appropriate for the functional analysis of the many genes that may reside in a typical genetically mapped locus of the order of 1 cM (31).

In the present study, the human CD83 molecule was analyzed in a physiological context using humanized transgenic mice generated by BAC-technology. BAC-hCD83 transgenic mice showed no extrinsic phenotype and developed a normal immune phenotype comparable to WT^tg^ mice. Interestingly, DCs generated from those mice showed a similar stimulatory capacity to prime T cells in an allogeneic setting as Wt mice derived cells did (Figure 3). With respect to the overall expression of human and murine CD83 in different organs, BAC-hCD83 transgenic mice expressed both predominantly in lymph node and spleen, whereas only low or no expression was found in all the other organs tested (Figure 4). This on the one hand clearly underlies the advantage of BAC technology to generate mouse models, where the gene of interest is expressed in a physiological manner (1). On the other hand this distribution of CD83 expression was expected when looking on a single cell level, since particularly activated DCs and B cells were the main source of murine and human CD83 (Figure 5). Interestingly, all DCs and B cells that expressed human CD83 were also positive for murine CD83, but not vice versa. This might be due to a competing expression caused by a limiting availability and usage of transcription factors involved in the regulation of the human and murine CD83. While the elements and transcription factors regulating human CD83 expression in DCs are well known (3), the murine CD83 promoter is not characterized. These data are in accordance with former reports, describing DCs and B cells as the predominant producers of membrane-bound and soluble CD83 (2, 4, 32, 33), and were confirmed by researchers using a CD83 reporter mouse, where a strong CD83 promoter activity was detected in lymph nodes and spleen (5). These authors described the resident B cell populations as a main source of CD83. Moreover, they found LPS-stimulated BMDCs to express unexpectedly high levels of CD83 *in vitro*, whereas immature DCs and CD11c- monocytes/macrophages did not. T cells only demonstrated an activation-dependent CD83 promoter activity, with 5–10% of naïve CD4^+^ and CD8^+^ T cells being CD83^+^, but up to 80% of CD4^+^ and 45% of CD8^+^ activated CD25^+^ T cells showed increased CD83 expression. Notably, we did not detect any human CD83 on the surface of either CD8^+^ or CD4^+^ T cells by flow cytometry, although stimulated CD8^+^ T cells expressed up to 9%, T conventional cells up to 34% and Tregs up to 45% murine CD83 (Figure 6). However, we found human CD83 on mRNA levels in stimulated CD8^+^ T cells of BAC-transgenic mice and low, but recurring intracellular human CD83 expression in CD4^+^ Tconv and Tregs (Figure 6). This is in accordance with flow cytometry data derived from human Tconv and Tregs, where we did not observe any CD83 surface-, but only intracellular expression, especially on stimulated human Tregs (unpublished data). Moreover, we found human CD83 to be immediately upregulated after stimulation on human Tregs (unpublished data) and huCD83-BAC^tg^-derived Tregs (5 h, Figure 6J), whereas Tconv cells showed highest human CD83 expression at later time points (16 h, Figure 6H) after stimulation.

As murine CD83 was described by several groups to contribute to their suppressive phenotype (7, 34), we next addressed the question, if Tregs derived from BAC-transgenic mice show a higher suppressive capacity and/or activation status. Indeed, especially Tregs derived from BAC-huCD83-77^tg^ mice, which expressed higher levels of human CD83 than BAC-huCD83-2^tg^ mice, showed a higher suppressive capacity in comparison to WT^tg^ mice derived cells (Figures 8, 9). Furthermore, also the production of pro-inflammatory cytokines was reduced in BAC-huCD83^tg^ derived cells. Moreover, we found higher levels of Treg-relevant gene transcripts including Foxp3, CTLA-4, and IL-10 in BAC-huCD83^tg^ mice, as well as an increased percentage of Foxp3^+^CD69^+^ and Foxp3^+^CD103^+^ Tregs (Figure 10), indicating that human CD83 plays an important role in the activation and suppressive function of Tregs. These data are in agreement with previous studies reporting (i) that continuous CD83 expression on activated human CD4^+^ T cells is important for their differentiation into iTregs (35) and (ii) that Lentivirus-mediated *in vitro* overexpression of murine CD83 in murine naïve CD4^+^CD25^−^ T cells leads to the conversion of Teff into T cells with suppressive capacity (34). A very recent study showed that Treg-intrinsic expression of CD83 is essential for Treg differentiation upon activation. Mice with Treg-intrinsic CD83 deficiency are characterized by a pronounced pro-inflammatory phenotype (6). The loss of CD83 expression by Tregs leads to the downregulation of Treg-specific differentiation markers and the induction of an inflammatory profile. In addition, Treg-specific conditional knockout mice showed aggravated autoimmunity and an impaired resolution of inflammation.

Since treatment with soluble CD83 prevented the development of EAE symptoms (20, 36), we investigated the role of human CD83 in the EAE model using our humanized BAC-huCD83^tg^ mice *in vivo* (Figure 7). Both, BAC-huCD83^tg^ and WT^tg^ mice develop classical EAE symptoms, however after the peak of disease BAC-huCD83^tg^ mice started to recover, while WT^tg^ mice did not, indicating that a better resolution of inflammation is induced in BAC-huCD83^tg^ mice. When analyzing the mice at the peak of disease; MOG-specific restimulation of BAC-huCD83^tg^ derived splenocytes was significantly impaired in comparison to WT^tg^ mice. Corresponding pro-inflammatory cytokines like IL-17 were reduced by trend, while levels of GM-CSF were significantly reduced in BAC-huCD83-77^tg^ mice upon antigen-specific MOG restimulation (Figure 7). As GM-CSF produced by T cells is necessary for the development of autoimmune CNS inflammation (37), this might contribute to the ameliorated disease course during resolution of inflammation. Next, cells involved in the autoimmune inflammatory processes in the CNS during EAE were analyzed and similar frequencies were detected in all treatment groups (Figure 7) and showed no differences. Also pro-inflammatory cytokines like IFN-γ and IL-17, which are associated with EAE, revealed equal levels in all animal groups. However, we detected higher Foxp3 expression in the CNS and splenic cells derived from BAC-huCD83^tg^ mice. Tregs play a critical role in the maintenance of peripheral immune tolerance and dysregulation of suppressive and migratory functions on Tregs have been linked to the pathogenesis of multiple sclerosis (MS) (38). For example, in patients with MS a decrease in the frequency of Tregs with reduced suppressive capacity was found (39). Furthermore, it was shown that in the EAE model Tregs play a major role in the recovery from EAE (40, 41). Interestingly, it was reported that an accumulation of Tregs in the CNS during the recovery phase of EAE has been a consistent finding in actively-induced models (42).

Taken together, we observed higher Foxp3 expression in the CNS and splenic cells derived from BAC-huCD83^tg^ mice and since Tregs derived from BAC-huCD83^tg^ mice had a more activated phenotype, these data suggests that CD83 not only plays a pivotal role in the suppressive function of Tregs *in vitro*, but also *in vivo*, by critically mediating the natural recovery from EAE. Moreover, these findings indicate an outstanding role for CD83 as a potent immune modulating molecule in EAE and multiple sclerosis as well as a potential candidate for Treg-targeted immunotherapy in autoimmune diseases.

## Ethics Statement

All animal experiments were performed in accordance with the European Communities Council Directive (86/609/EEC), and were approved by the local ethics committee Government of Middle Franconia, Germany.

## Author Contributions

IK, EZ, and AS designed the experiments, analyzed, interpreted the data, and wrote the manuscript. RN generated the BAC-transgenic mice. EZ, JM, AD, AW, LS, and CK performed experiments and analyzed the data. All authors approved the final version of the paper.

### Conflict of Interest Statement

The authors declare that the research was conducted in the absence of any commercial or financial relationships that could be construed as a potential conflict of interest.
