# Supplementary material for: Endogenous Expression of the Human CD83 Attenuates EAE Symptoms in Humanized Transgenic Mice and Increases the Activity of Regulatory T Cells
Source: Front Immunol. 2019 Jun 25;10:1442. doi: 10.3389/fimmu.2019.01442 (PMC6603205; doi:10.3389/fimmu.2019.01442)
Supplement: Supplementary file 1 [file Data_Sheet_1.docx]

Supplementary Material

## Supplementary Figure 1


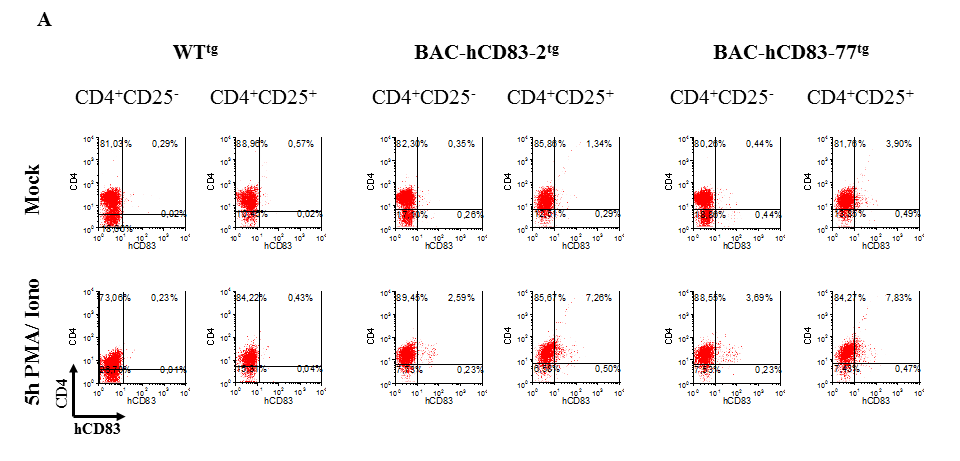


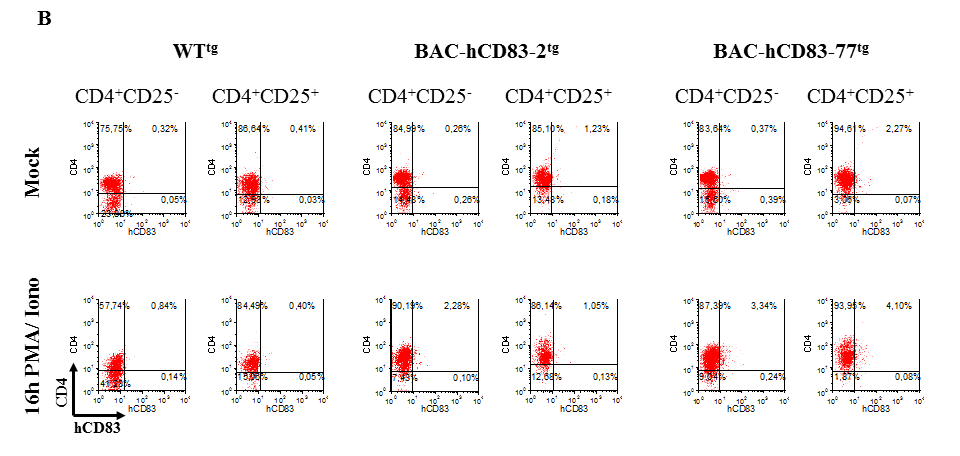


**Supplementary Figure 1.** CD4^+^CD25^-^ and CD4^+^CD25^+^ cells derived from WT^tg^, BAC-hCD83-2^tg^, or BAC-hCD83-77^tg^ mice were isolated from whole spleen cells using MACS technology. T cells were either left unstimulated (mock) or stimulated with 50 ng/ml PMA plus 500 ng/ml Ionomycin (PMA/Iono) for 5 (A) or 16 (B) hours before they were analyzed by flow cytometry. Dot plots of intracellular stainings on hCD83 of CD4^+^CD25^-^ and CD4^+^CD25^+^ MACS-sorted T cells. One representative experiment out of three is shown.
